# Neutrophil Extracellular Traps Effectively Control Acute Chikungunya Virus Infection

**DOI:** 10.3389/fimmu.2019.03108

**Published:** 2020-01-31

**Authors:** Carlos H. Hiroki, Juliana E. Toller-Kawahisa, Marcilio J. Fumagalli, David F. Colon, Luiz T. M. Figueiredo, Bendito A. L. D. Fonseca, Rafael F. O. Franca, Fernando Q. Cunha

**Affiliations:** ^1^Department of Pharmacology, School of Medicine of Ribeirao Preto, University of São Paulo, Ribeirao Preto, Brazil; ^2^Department of Biochemistry and Immunology, School of Medicine of Ribeirao Preto, University of São Paulo, Ribeirao Preto, Brazil; ^3^Virology Research Center, School of Medicine of Ribeirao Preto, University of São Paulo, Ribeirao Preto, Brazil; ^4^Department of Virology and Experimental Therapy, Institute Aggeu Magalhaes, Oswaldo Cruz Foundation, Recife, Brazil

**Keywords:** NETs, Chikungunya, neutrophils, viral infection, innate response

## Abstract

The Chikungunya virus (CHIKV) is a re-emerging arbovirus, in which its infection causes a febrile illness also commonly associated with severe joint pain and myalgia. Although the immune response to CHIKV has been studied, a better understanding of the virus-host interaction mechanisms may lead to more effective therapeutic interventions. In this context, neutrophil extracellular traps (NETs) have been described as a key mediator involved in the control of many pathogens, including several bacteria and viruses, but no reports of this important protective mechanism were documented during CHIKV infection. Here we demonstrate that the experimental infection of mouse-isolated neutrophils with CHIKV resulted in NETosis (NETs release) through a mechanism dependent on TLR7 activation and reactive oxygen species generation. *In vitro*, mouse-isolated neutrophils stimulated with phorbol 12-myristate 13-acetate release NETs that once incubated with CHIKV, resulting in further virus capture and neutralization. *In vivo*, NETs inhibition by the treatment of the mice with DNase resulted in the enhanced susceptibility of IFNAR^−/−^ mice to CHIKV experimental acute infection. Lastly, by accessing the levels of MPO-DNA complex on the acutely CHIKV-infected patients, we found a correlation between the levels of NETs and the viral load in the blood, suggesting that NETs are also released in natural human infection cases. Altogether our findings characterize NETosis as a contributing natural process to control CHIKV acute infection, presenting an antiviral effect that helps to control systemic virus levels.

## Introduction

The Chikungunya virus (CHIKV) is a single-stranded RNA virus that is transmitted to humans by the bite of the infected mosquitoes from the *Aedes* family (1). This virus was first isolated from a patient in Tanzania in 1952, and since then, reports of this infection have been described on all continents, mainly in tropical regions such as Africa, South Asia, and both South and Central America (2, 3). The symptoms typically include fever, headache, and a papular or maculopapular rash during the acute stage. In most cases, the disease is self-limiting; however, some patients can manifest chronic and debilitating arthralgia, which can last for months and even years (1).

After inoculation by a mosquito, CHIKV infects the resident cells—including fibroblasts, macrophages, and endothelial cells—and starts to proliferate (4). These cells recognize the virus via innate receptors and produce proinflammatory mediators, recruiting and activating immune cells to eliminate the pathogen (5). Among these cells, the monocytes and the dendritic cells have been widely studied; however, the role of the neutrophils is still poorly understood (6). During virus infection, the neutrophils are recruited to the inflammation site through the production of chemoattractant molecules by the resident cells, such as CXCL1 and CXCL2 (7, 8). Once in the tissue, the emigrated neutrophils start to produce reactive oxygen species (ROS) and other cytotoxic mediators, which may dampen the virus infection (9). It has become clear in the literature that the neutrophils are able to release neutrophil extracellular traps (NETs), which are a sticky web of DNA conjugated with antimicrobial enzymes (such as myeloperoxidase and histones), resulting in the capture and the killing of different pathogens, including viruses (9, 10).

The process of NETs production, denominated NETosis, has been widely studied over the past few years. In general, the process starts with neutrophil activation by the pattern recognition receptors (PRR), followed by ROS production. This production leads to the induction and the activation of protein arginine deiminase 4, an intracellular protein responsible for histone citrullination, which results in chromatin decondensation (11). During a viral infection, such as those caused by the respiratory syncytial virus (RSV) and the HIV-1, NETosis can be induced through the recognition of viral antigens by the PRR, such as the Toll-like receptor (TLR) 4, 7, or 8. Once released, the NETs are responsible for virus capture and inactivation; however, if excessive, the NETs can also induce organ damage (12). In a CHIKV infection, the neutrophils are recruited and start to produce type I interferon (IFN) to eliminate the virus (13), but there are no reports that demonstrate the role of the NETs in CHIKV killing. Thus, the aim of the present study was to demonstrate whether the NETs could be induced by a CHIKV infection, the possible mechanism that triggers their release, and their physiological relevance.

Here we found that mouse and human neutrophils release NETs after incubation with CHIKV, and in mice, NETs release occurs through a TLR7- and ROS-dependent mechanism. Moreover, the NETs were able to neutralize a CHIKV infection *in vitro*, and this effect was abolished after the DNase treatment. *In vivo*, NETs inhibition led to an earlier increase in the viral load and the enhanced susceptibility of the infected IFNAR^−/−^ mice. Lastly, we found that during the acute phase, the patients with confirmed CHIKV infection had an increased abundance of NETs-associated MPO–DNA complexes in their serum, which correlated with a higher viral load. Altogether these findings suggest an antiviral role for NETs during a CHIKV acute infection.

## Materials and Methods

### Virus, Drugs, and Cells

CHIKV was isolated from a positive serum sample at Oswaldo Cruz Foundation, Fiocruz/Recife, Brazil, by amplification in Vero E6 cells. Briefly, 50 μl of real-time RT-PCR (rRT-PCR)-positive serum from a male adult with CHIKV symptoms was incubated for 1 h at room temperature on Vero E6 cell monolayers previously grown with Dulbecco's modified Eagle medium high glucose (Thermo Fisher Scientific) supplemented with 10% fetal bovine serum (Gibco). The cells were then further incubated at 37°C until a cytopathic effect was apparent.

Following isolation, virus stocks were prepared in Vero E6 cells and stored at −80°C for virus titration by assaying for plaque-forming units (PFU) as previously described (14). The Zika virus (ZIKV) strain PE243 (ZIKV/H.sapiens/Brazil/PE243/2015) and the dengue-2 (DENV2) strain 16681 were both prepared in Vero E6 cells as previously established in our laboratory (14).

Treatment with apocynin (Sigma-Aldrich) was performed as previously described (15). Briefly, the neutrophils were treated with 300 μM apocynin for 30 min at 37°C and then stimulated with CHIKV. For NETs release, 2 × 10^6^ neutrophils were stimulated with 100 nM PMA (Sigma-Aldrich) for 4 h at 37°C, followed by treatment with the medium or 5 mg/ml rhDNase (Roche) for 2 h at 37°C.

### Mice

C57BL/6 or 129S6/SVEV (wild type, WT) mice were obtained from the animal facility of the University of São Paulo, São Paulo, Brazil. The TLR3^−/−^, TLR3/7/9^−/−^ (triple knockout), TLR9^−/−^, and IFNAR^−/−^ mice were purchased from Jackson Laboratory. This study was approved by the Ethics Committee on the Use of Animals (CEUA) of the University of São Paulo (protocol number 0005/2017).

### NETs Quantification (MPO–DNA PicoGreen)

The mouse or human neutrophil isolation was performed as previously described (16, 17). A total of 2 × 10^6^ neutrophils were incubated with CHIKV, ZIKV, or DENV2 for different times in a volume of 300 μl at 37°C. MOCK (Vero E6 media without virus) was used as negative control. NETs quantification was performed as previously described (18). Briefly, an anti-MPO antibody bound to a 96-well flat-bottom plate captured the enzyme MPO (Thermo Fisher Scientific), and the amount of DNA bound to the enzyme was quantified using the Quant-iT™ PicoGreen® kit (Invitrogen) according to the manufacturer's instructions. Fluorescence intensity (emission at 488-nm wavelength) was quantified in a FlexStation 3 Microplate Reader (Molecular Devices, CA, USA).

### Immunofluorescence of NETs

A total of 5 × 10^4^ isolated neutrophils were attached on a slide coated with poly-*D*-lysine (Sigma-Aldrich) and incubated with CHIKV. After 4 h of incubation, the slides were washed with phosphate-buffered solution (PBS) and fixed with 4% paraformaldehyde for 30 min. The samples were blocked with a PBS/BSA 2% solution (Sigma-Aldrich) for 2 h at room temperature and incubated with primary antibodies against histone H3 citrulline R17+R2+R8 (ab5103, Abcam, 1:500), murine polyclonal anti-CHIKV antibodies (obtained from Dr. Figueiredo's lab, 1:100), and anti-Ly6G antibodies (16-9668-82, Invitrogen, 1:50) overnight at 4°C. After washing with PBS, anti-rabbit Alexa Fluor 488 (1:1000, Molecular Probes), anti-rat Alexa Fluor 594 (1:100, Molecular Probes), and/or anti-mouse Alexa Fluor 594 (1:200, Molecular Probes) were incubated for 2 h at room temperature. The slides were counterstained with DAPI (P36935, Molecular Probes), and the images were acquired with a Leica TCS SP5-AOBS microscope (Leica Microsystems, Mannheim, Germany).

### ROS Evaluation

ROS was measured as previously described (19). Briefly, the isolated neutrophils (2 × 10^5^) were incubated with CHIKV and 1,000 μM luminol (5-amino-2,3-dihydro-1,4-phthalazinedione; Sigma–Aldrich, St. Louis, MO, USA). The neutrophils incubated with MOCK and luminol were used as a negative control. The chemiluminescence reaction was monitored by a Flexstation 3 Microplate Reader (Molecular Devices, California, USA) for 2 h at 37°C. The results are expressed as the area under the curve (AUC) of the time-course.

### Viability Assay

Viability was measured using Fixable Viability Dyes (Invitrogen) according to the manufacturer's instructions.

### *In vivo* Infection

The IFNAR^−/−^ mice were intraperitoneally infected with 30 PFU of CHIKV and treated subcutaneously with 10 mg/kg rhDNase (Roche) or saline every 12 h until the end of the experiment. Peripheral blood was collected from the orbital sinus every 24 h for the NETs and viral load quantification.

### Patient Samples

The suspected Chikungunya clinical cases were diagnosed by rRT-PCR from the serum samples forwarded to the Arbovirus Reference Laboratory at the Oswaldo Cruz Foundation, Fiocruz/Recife, Brazil. Real-time PCR protocol was employed as previously described (20). The blood samples were collected from different locations in the state of Pernambuco, northeastern Brazil, from patients presenting with rash, arthralgia, and/or fever. Samples from healthy donor were collected and stored at −80°C until use. The samples were collected after written informed consent was given by the patients and the healthy donors. This study was approved by the Oswaldo Cruz Foundation Ethics Committee (protocol number 2.566.608).

### CHIKV Quantitative Real-Time RT-PCR

The viral RNA from CHIKV patients was isolated using a QIamp Viral RNA Mini Kit (Qiagen) according to the manufacturer's protocol. For the CHIKV RNA quantification, the viral RNA was amplified (primer F sequence AAAGGGCAAACTCAGCTTCAC and primer R sequence GCCCTGGGCTCATCGTTATTC) and detected using a fluorescent probe (CHIKV FAM, sequence CGCTGTGATACAGTGGTTTCGTGTG) with the QuantiNova Probe RT-PCR Kit QuantiNova Kit (Qiagen), according to the manufacturer's protocol, in a one-step real-time PCR format (Applied Biosystems).

### Statistical Analysis

The statistical analyses were performed using GraphPad-Prism 6 (GraphPad Software Inc., San Diego CA, USA). The results were expressed as mean values and their standard deviations. For a comparison between multiple groups, the analysis of variance was used with Bonferroni's comparison test. To compare the median between the two groups, the Mann–Whitney *U*-test was used. The survival rate was expressed as the percentage of live animals, and the Mantel–Cox log–rank test was used to determine the differences between the survival curves. The correlation between two data points was performed by the Spearman rank correlation test.

## Results

### Mouse-Isolated Neutrophils Release NETs Following a CHIKV but Not a ZIKV and a DENV Infection

**Figure 1:**
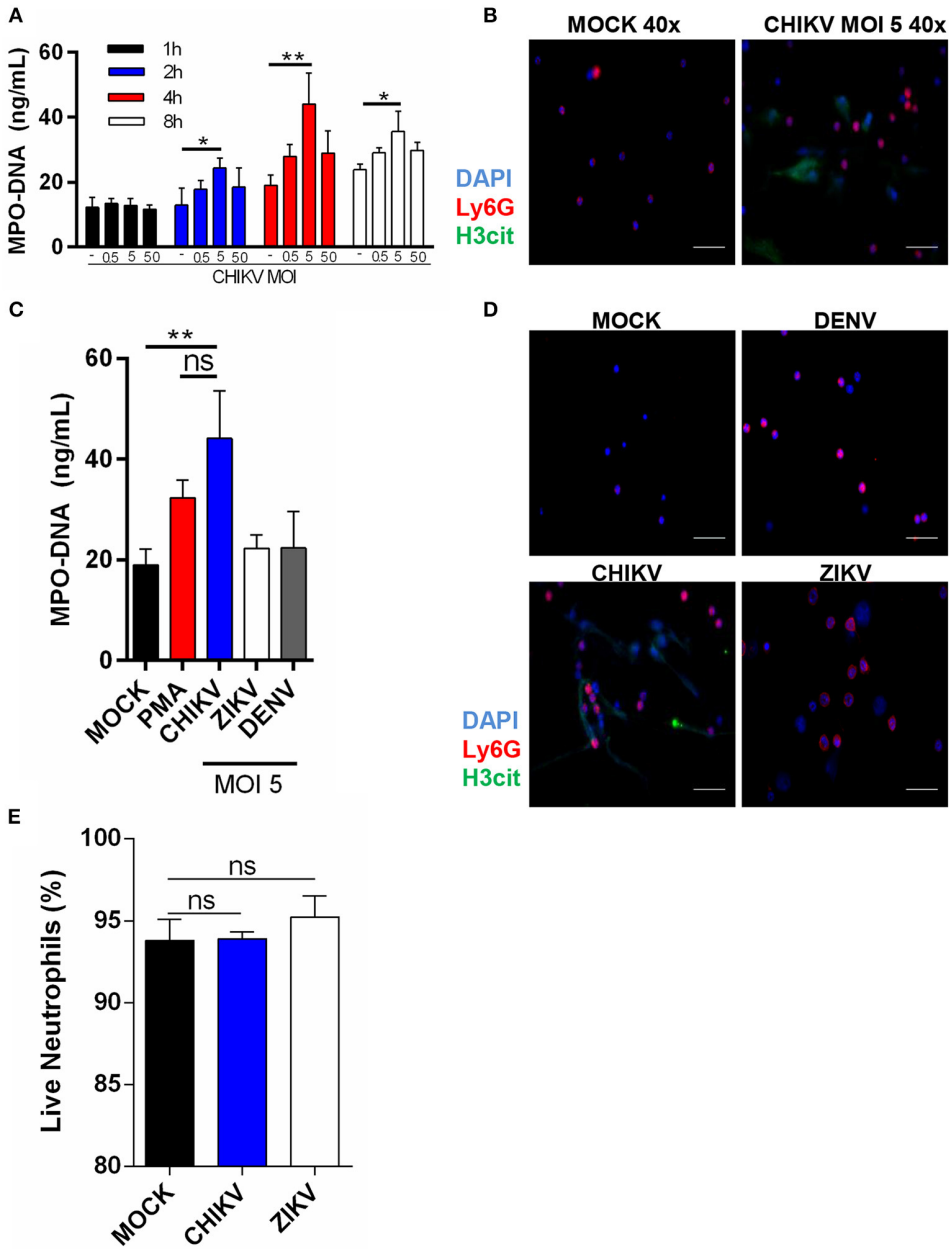
Isolated neutrophils release NET following CHIKV, but not ZIKV or DENV infection. **(A)** Quantification of NETs by MPO–DNA PicoGreen in the supernatant of isolated mouse neutrophils incubated with CHIKV at different MOIs for 1, 2, 4, and 8 h. **(B)** Representative image of immunofluorescence of mouse neutrophils incubated with CHIKV for 4 h. *Bars* = 50 μm. Magnification ×40. Cells were stained with DAPI (*blue*), anti-Ly6G (*red*), and anti-H3 citrulline (*green*). **(C)** Quantification of NET by MPO–DNA PicoGreen in the supernatant of mouse neutrophils incubated with PMA (100 nM), CHIKV, ZIKV, or DENV (MOI = 5) for 4 h. **(D)** Representative immunofluorescence image of mouse neutrophils incubated with CHIKV, ZIKV, or DENV (MOI = 5) for 4 h. *Bars* = 50 μm. Magnification ×40. Cells were stained with DAPI (*blue*), anti-Ly6G (*red*), and anti-H3 citrulline (*green*). **(E)** Percentage of live neutrophils incubated with MOCK control, CHIKV, or ZIKV for 4 h. Data are presented as mean ± SD (*n* = 3 per group); *p < 0.05 and **p < 0.01, assessed by two-way **(A)** or one-way ANOVA **(C,E)** with Bonferroni's comparisons test. Representative results of two experiments performed independently.

To identify whether CHIKV could induce NETs release, we first incubated the murine neutrophils with CHIKV at different multiplicities of infection (MOI = 0.5, 5, or 50) for 1, 2, 4, or 8 h. Following the virus adsorption, the infected cell supernatants were harvested, and the NETs-associated MPO–DNA free complexes were quantified by the MPO–DNA PicoGreen. The peak of the NETs release was detected at 4 h post-infection (hpi) with MOI = 5 (Figure 1A). Although the peak of the NETs production was similar at 8 hpi, negative control cells were also activated at later time points (8 hpi), probably as a consequence of cell stress. Therefore, we performed the following experiments utilizing MOI = 5 and 4 h of incubation. To further confirm NETs release, we performed an immunofluorescence assay from the virus-stimulated neutrophils in which the NETs were identified as extracellular complexes that were simultaneously costained for free DNA (DAPI) and citrullinated histone H3 (H3cit) (Figure 1B and Supplementary Figure 1A). Thus, we also confirmed by immunofluorescence that CHIKV (MOI = 5) stimulates NETs release at 4 hpi.

To confirm whether NETs release was a specific neutrophil response to CHIKV—and not a general response against other well-known arboviruses—we incubated the neutrophils with ZIKV or DENV2 (MOI = 5 and 4 h of incubation). Indeed we observed that only CHIKV was able to induce NETs release as assessed by MPO–DNA PicoGreen (Figure 1C) and immunofluorescence (Figure 1D). Currently, two NETosis pathways are described in the literature: lytic and non-lytic NETosis (12). We observed many “intact” neutrophils in our images (Figures 1B,D), so we wondered if NETosis induced by the CHIKV was leading to neutrophil death. Curiously, we observed no cell death after the incubation with CHIKV, suggesting that this virus leads to non-lytic NETosis (Figure 1E).

### CHIKV-Induced NETosis Occurs Through a TLR7- and ROS-Dependent Mechanism

**Figure 2:**
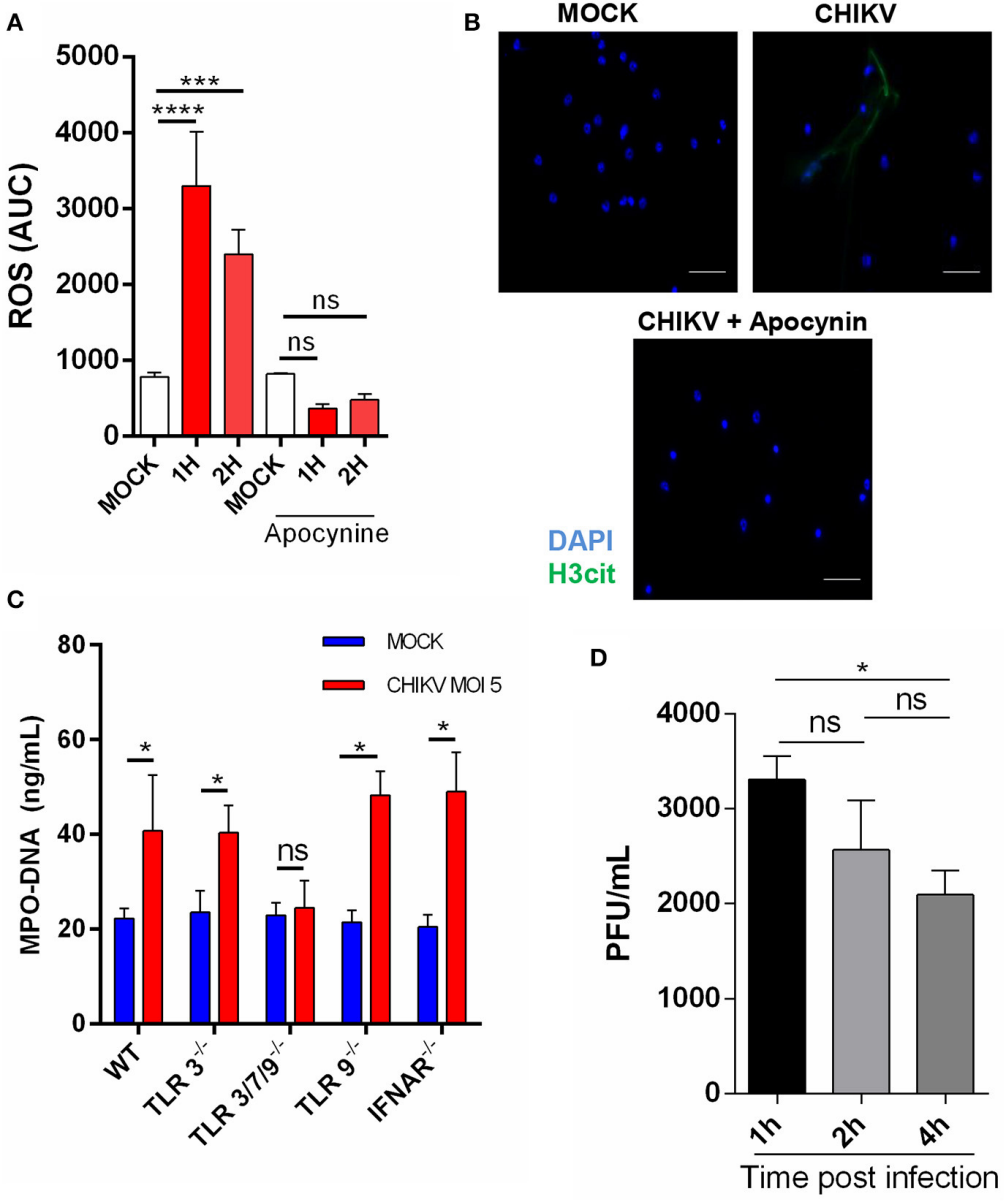
CHIKV triggers TLR7- and ROS-dependent NETosis. **(A)** Production of ROS by mouse neutrophils incubated with CHIKV (MOI = 5) and treated or not with apocynin (300 μm) for 30 min prior to CHIKV incubation. **(B)** Representative image of an immunofluorescence assay of mouse neutrophils incubated with CHIKV (MOI = 5) and treated or not with apocynin (300 μM) for 30 min prior to CHIKV stimulation. *Bars* = 50 μm. Magnification ×40. Samples were stained with DAPI (*blue*) and anti-H3 citrulline (*green*). **(C)** Quantification of NETs by MPO–DNA PicoGreen in the supernatant of TLR3^−/−^, TLR3/7/9^−/−^, TLR9^−/−^, and IFNAR^−/−^ mouse-isolated neutrophils after incubation with CHIKV (MOI = 5 for 4 h). **(D)** RT-qPCR for CHIKV from neutrophils incubated with virus stocks after 1, 2, and 4 h. Data are presented as mean ± SD (*n* = 4 per group); *p < 0.05, ***p < 0.001, and ****p < 0.0001, assessed by one-way **(A,D)** or two-way ANOVA **(C)** with Bonferroni's comparisons test. Representative results of two experiments performed independently.

Although the intracellular mechanisms of NETosis are still not completely elucidated, a classical hallmark of its activation is ROS production, which has also been described to be induced during virus infection (11, 21). To explore this effect, we performed a time-course CHIKV infection experiment in which the ROS peak was observed at 1 hpi and remained constant at 2 hpi. To confirm the correlation of ROS induction by CHIKV and NETosis, we employed an NADPH oxidase inhibitor (apocynin) that directly blocks ROS production. After the treatment with apocynin, we observed a strong inhibition of ROS production in the cells infected with CHIKV (Figure 2A). Additionally, the apocynin treatment was also able to inhibit the CHIKV-induced NETosis (Figure 2B), suggesting that the CHIKV-induced NETosis occurs dependently on ROS production.

As previously described, the PRR are activated by viruses, triggering NETosis (21, 22). Thus, to identify which receptor could be triggered by the CHIKV, we isolated the neutrophils from different knockout mouse strains and performed the infection assays as described above. We observed that the neutrophils derived from the TLR3/7/9^−/−^ mice (TLR triple knockout) were unable to release NETs (Figure 2C). Next, we assessed the participation of each TLR separately. We found that the TLR3^−/−^ and TLR9^−/−^ mouse neutrophils were able to respond to the CHIKV infection by inducing NETs release, which suggests that TLR7, but not TLR3^−/−^ and TLR9^−/−^, contributes to the CHIKV NETs induction (Figure 2C). Moreover, similar to WT, the neutrophils derived from TLR3^−/−^, TLR3/7/9^−/−^, and TLR9^−/−^ showed no cell death after incubation with CHIKV (Supplementary Figure 1B). TLR7 is an endolysosome able to recognize single-stranded RNA virus and trigger NETosis (21). Therefore, we next evaluated if CHIKV is able to infect the neutrophils, allowing TLR7 recognition. We performed neutrophil incubation with CHIKV and measured the viral load in the cells. We found an increased viral load after 1 h of incubation, which decreased on the next hours (Figure 2D), suggesting that CHIKV can infect the neutrophils and be recognized by TLR7, thus triggering NETosis.

### NETs Release Contributes to Virus Neutralization

**Figure 3:**
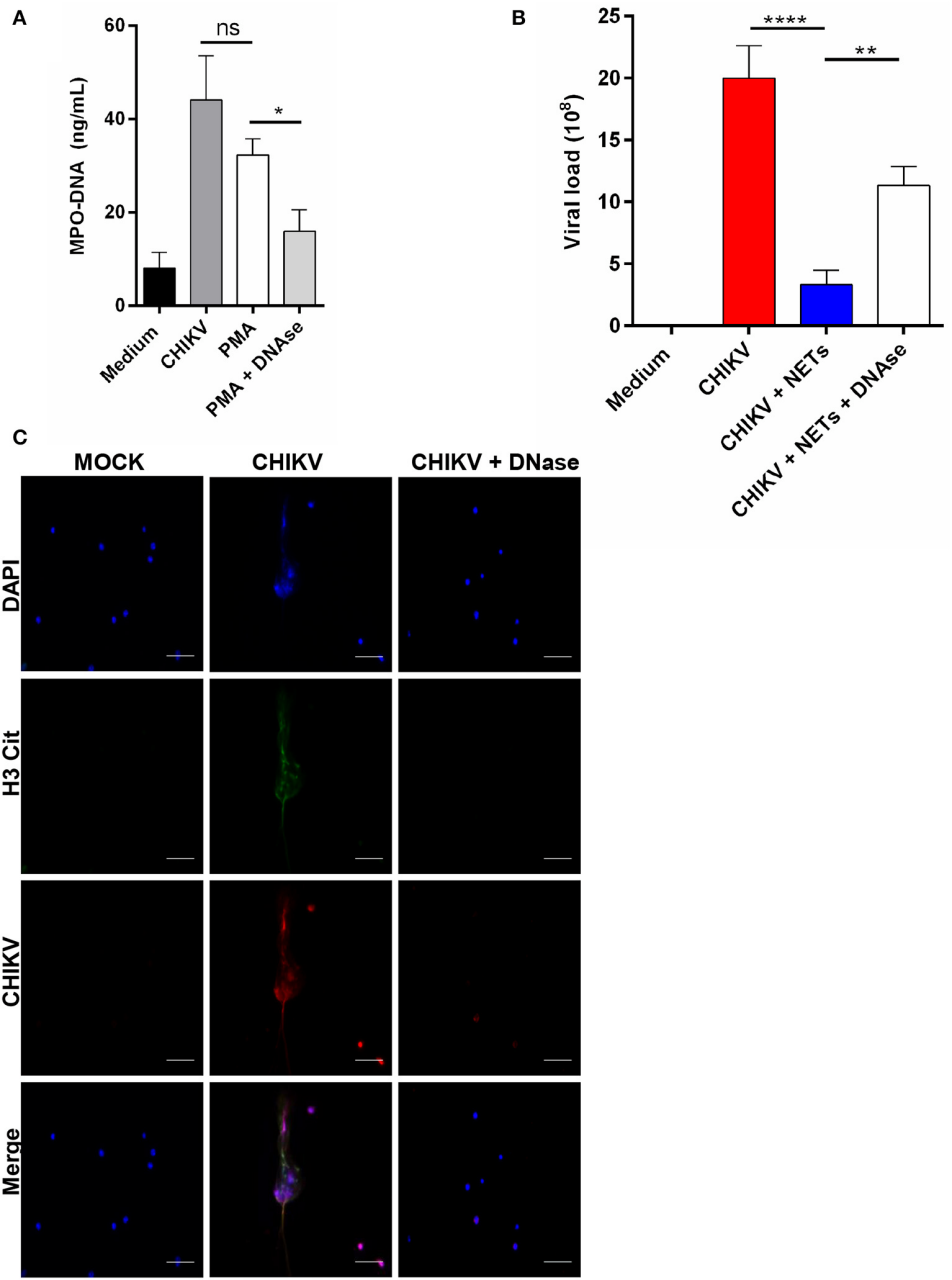
NETs release contributes to virus neutralization. **(A)** Quantification of NETs by MPO–DNA PicoGreen in the supernatant of mouse neutrophils incubated with medium, CHIKV, PMA (100 nM), and DNase (5 mg/ml) for 4 h. **(B)** Viral load quantification by PFU assay of CHIKV virus stocks incubated with medium, NETs, or NET predigested with DNase for 2 h. **(C)** Representative immunofluorescence image of mouse neutrophils incubated with CHIKV (MOI = 5 for 4 h). *Bars* = 50 μm. Magnification ×40. Cells were stained with DAPI (*blue*), anti-H3 citrulline (*green*), and anti-CHIKV (*red*). Data are presented as mean ± SD (*n* = 4 per group); *p < 0.05, **p < 0.01, and ****p < 0.0001, assessed by one-way ANOVA with Bonferroni's comparisons test. Representative results of two experiments performed independently.

Since the antiviral effects of the NETs were described by others (12), we next evaluated whether the NETs were able to control the CHIKV infection *in vitro*. First, the NETs were generated by stimulating isolated mouse neutrophils with PMA at 100 nM for 4 h. Clearly, we observed that the PMA treatment results in NETs release (Figure 3A). Next, the PMA-stimulated cell supernatants were incubated with either DNase (5 mg/ml) or medium for 2 h, and the NETs were further quantified. We observed that the treatment with DNase reduced the amount of MPO–DNA free complexes (Figure 3A). Thus, to evaluate the direct antiviral effects of the NETs, the CHIKV was incubated with PMA neutrophil-stimulated supernatants (previously treated or not with DNase) for 2 h at 37°C, and the infectivity was evaluated by PFU assay. Here we observed a significant reduction in the CHIKV infection capacity after incubation with the NETs, and the infectivity was restored when the NETs were pre-digested with DNase (Figure 3B). Furthermore, to evaluate whether the NETs could directly interact with the CHIKV, we incubated isolated murine neutrophils with CHIKV and stained both the NETs and the virus (Figure 3C). We observed the presence of the virus aggregates that colocalized with free DNA (DAPI) and citrullinated histone H3 (H3cit), suggesting that the NETs were effective in capturing the virus particles.

### DNase Treatment Increases Viral Load and Susceptibility *in vivo*

**Figure 4:**
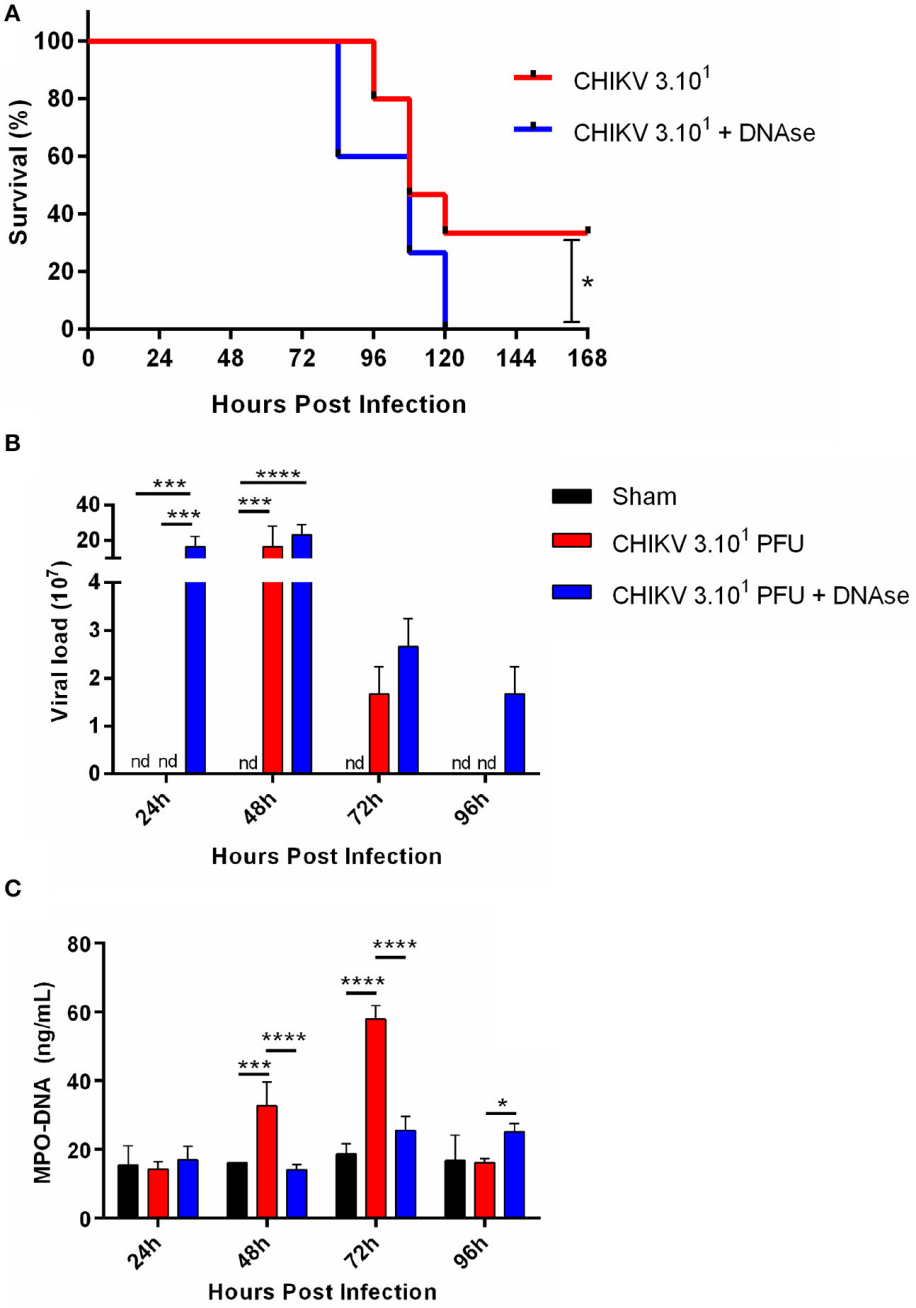
DNase treatment increases viral load *in vivo*. **(A)** Survival of IFNAR^−/−^ mice infected i.p. with 30 PFU of CHIKV and treated with saline or DNase (10 mg/kg, s.c., every 12 h). **(B)** Quantification of NETs by MPO–DNA PicoGreen and **(C)** viral load in the plasma of infected IFNAR^−/−^ treated with saline or DNase. Data are mean ± SD (*n* = 10 per group); *p < 0.05, ***p < 0.001, and ****p < 0.0001, assessed by Mantel–Cox log–rank test **(A)** or two-way ANOVA with Bonferroni's comparisons test **(B,C)**. Representative results of three experiments performed independently.

As demonstrated by others, the IFNAR^−/−^ mice, being a reliable model for *in vivo* experimentation, are highly susceptible to CHIKV infection (23). Applying this same approach, we established a semilethal *in vivo* challenge model by inoculating the IFNAR^−/−^ mice with 30 PFU of CHIKV, which resulted in 30% survival rate. In our model, the infected mice began to die at ~4 days post-infection (dpi) (Figure 4A). Thus, to demonstrate the role of NETs *in vivo*, the CHIKV-infected animals were treated or not with DNase (10 mg/kg every 12 hpi), and the survival rates were checked daily. Interestingly, we observed that the DNase treatment resulted in an anticipated mortality and an increased susceptibility to CHIKV infection (*p* = 0.0352) when compared to the untreated group of mice (Figure 4A).

Moreover, the DNase-treated animals had increased viremia, detected at 1 dpi, which starts to decrease in the following days. The DNase treatment also resulted in extended virus detection *in vivo*, where the CHIKV was still detected at 4 dpi. Instead, in the saline-treated animal group, the CHIKV replication was delayed since we detected virus at 2 dpi, turning undetectable at 4 dpi (Figure 4B). Concomitantly with virus detection in the blood, the serum NETs levels were increased at 2 dpi, reaching a peak at 3 dpi in the saline-treated group. On the other hand, the NETs levels remained lower after the DNase treatment throughout the whole experiment period (Figure 4C). Altogether these data suggest that the NETs are important to control the CHIKV acute replication *in vivo*.

### Human Neutrophils Are Responsive to the CHIKV Infection by Inducing NETs Release

**Figure 5:**
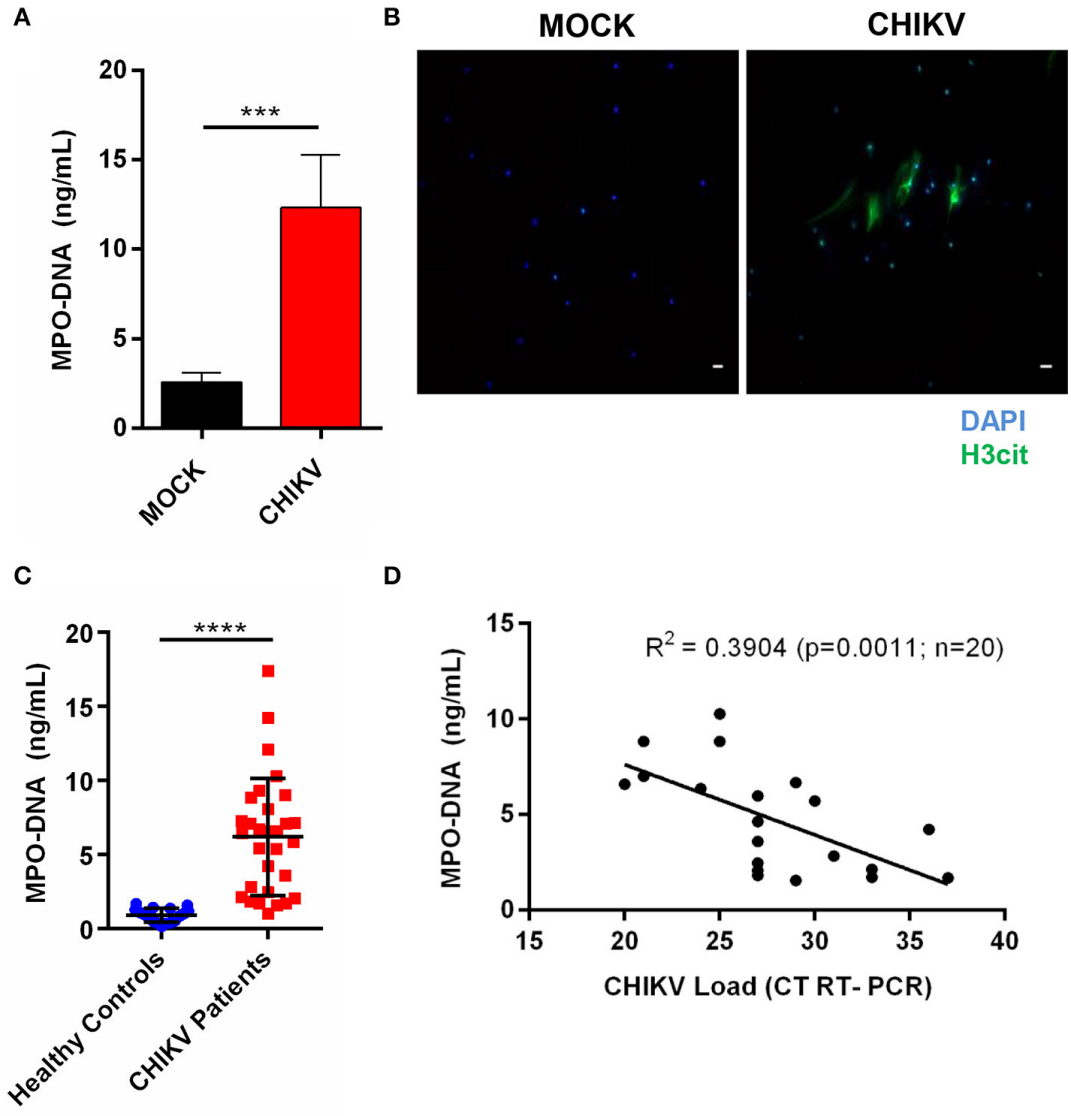
CHIKV induces NETosis in human neutrophils, which is correlated with viral load in clinical samples. **(A)** Quantification of NETs by MPO–DNA PicoGreen of human neutrophils incubated with CHIKV (MOI = 5 for 4 h). **(B)** Representative immunofluorescence image of human neutrophils incubated with CHIKV (MOI 5 for 4 h). *Bar* = 50 μm. Magnification ×20. Samples were stained with DAPI (*blue*) and anti-H3 citrulline (*green*). **(C)** Viral load in the serum of acute CHIKV patients assessed by rRT-PCR. **(D)** Spearman's correlation between NETs concentration and viral load in the serum of acute CHIKV patients. Data are presented as mean ± SD (*n* = 3 per group); ***p < 0.001 and ****p < 0.0001, assessed by one-way ANOVA with Bonferroni's comparisons test **(A)**, Mann–Whitney test **(C)**, and Spearman's correlation **(D)**.

Human neutrophils have also been described to release NETs after virus incubation (21). Thus, to confirm whether the CHIKV infection could induce NETs release in human neutrophils, we applied the same conditions as described above for the experimental *in vitro* infection assays (4 h MOI = 5). Here our data demonstrate that the CHIKV incubation leads to an increase in the NETs-associated free MPO–DNA complexes, as quantified by the MPO–DNA PicoGreen (Figure 5A). The NETs release was also confirmed by immunofluorescence in which free DNA-associated (DAPI) citrullinated histone H3 (H3cit) complexes were observed after the incubation with CHIKV (Figure 5B).

Since the NETs levels have already been associated with disease prognosis during other viral infections (24, 25), we hypothesized that the NETs would be induced in patients with a confirmed CHIKV infection. To assess this, the serum samples from patients with a confirmed CHIKV acute infection, obtained during a recent outbreak in northeastern Brazil, and from healthy control donors (individuals with no infection) were used for the NETs quantification by the MPO–DNA PicoGreen. By doing this, we observed increased levels of the NETs in the serum from the acutely infected patients compared to those from the healthy controls (Figure 5C). Additionally, we performed a correlation test analysis between the NETs levels and the CHIKV RNA levels based on the cycle threshold values (assessed by semiquantitative rRT-PCR). Thus, albeit we were not able to demonstrate the amount of infectious particles in the blood, we observed a direct significant correlation (*p* = 0.0011) between the NETs levels and the CHIKV RNA levels (viremia), demonstrating that the CHIKV high viral load was also associated with the high NETs levels in humans (Figure 5D).

## Discussion

The arbovirus infections are increasing each year due to globalization, global warming, virus adaptation to new vectors, and other factors, affecting millions of people and causing serious problems for public health. Importantly, viral persistence induces chronic arthralgia, which is the main clinical consequence for the CHIKV patients (26). Thus, understanding the mechanisms by which the mammalian hosts deal with CHIKV could allow the development of therapeutic strategies aimed at enhancing the early host clearance of the virus. Here we report that the CHIKV infection induces ROS- and TLR7-dependent NETosis, which in turn helps to control a viral infection and consequently protects the host.

The neutrophils are the first leukocyte subtype to infiltrate the infected sites; however, until now, only a few studies have demonstrated the role of these cells during viral infections (6). In zebrafish experimentally infected with CHIKV, the neutrophils are an important source of type I IFN, and the depletion of these cells leads to an increase in both the disease score and the viral load, demonstrating the importance of these cells for the control of this virus (13). However, even after 15 years since the NETs were first described, the relevance of this mechanism during virus infections, especially by arboviruses, remains poorly understood (12). Here we found that the CHIKV, but not the ZIKV and the DENV2, was capable of inducing the NETs production in isolated mouse and human neutrophils (Figures 1C,D, 5A). Thus, our results reinforce the importance of studying the influence of the NETs in other Alphavirus infections, such as the Mayaro virus and the Ross River virus. Similar to our results, the Sendai virus, the HIV-1, the RSV, and the Hantavirus stimulate NETs release by isolated neutrophils (21, 22, 27, 28). In the same way, Moreno-Altamirano et al. (29) also demonstrated that the DENV2 was not able to induce the NETs (29), but another study controversially showed that this virus, in fact, triggered NETosis (30). Clearly, the experimental differences may explain this apparent contradiction. However, in patients, it is suggested that the proinflammatory cytokines, such as IL-8 and TNF-α, produced during DENV infection could result in the NETs production, suggesting that arbovirus-stimulated NETosis can also be induced indirectly (31).

We also demonstrated that the CHIKV-triggered NETosis is dependent on TLR7 activation and ROS production (Figures 2B,C). Similarly, the HIV-1 was able to induce NETs production in human neutrophils, and the pharmacological inhibition of TLR7 and TLR8 or ROS production resulted in NETs ablation (21). This might be explained by the molecular structure of both viruses, which consist of a single-stranded RNA, allowing TLR7 recognition. Similarly, the NETosis triggered by RSV was ablated when the TLR4 or ROS production was blocked (22), demonstrating the importance of PRR recognition and ROS signaling in the virus-induced NETs. Together our results and those mentioned studies support that the neutrophils are able to recognize viruses, triggering the NETosis process. TLR3 was previously described to contribute for CHIKV control (32). Due to the absence of a specific *knockout* for TLR7 in our mouse facility, we had to use a triple *knockout* for TLR3/7/9 to relate this receptor with NETosis induction. Even though TLR3^−/−^- and TLR9^−/−^-derived neutrophils showed NETs production after a CHIKV incubation, we cannot exclude a synergic pathway involving these receptors during the NETosis induced by this virus.

Since their discovery, the NETs have been demonstrated to capture and kill different pathogens (33). Thus, to demonstrate the antiviral activity of the NETs, we incubated CHIKV stocks with neutrophil supernatants containing a high concentration of pre-induced NETs. By doing so, we observed a decreased capacity of infection, which was partially restored when the NETs were pre-digested with DNase (Figures 3B,C). In agreement with our findings, the NETs were also able to capture RSV and to inhibit its infection in the A549 lung epithelial cells (34) as well as HIV-1 in the CD4^+^ T cells (35), clearly demonstrating their antiviral role.

Upon extending these findings, we observed that the treatment with DNase increases the susceptibility to CHIKV infection in IFNAR^−/−^ mice, which also demonstrated increased viral load in the blood, suggesting a protective role of the NETs *in vivo* (Figures 4A,B). Curiously, at 24 hpi, the DNAse treatment resulted in an increased viral load despite the fact that elevated NETs levels in the blood were observed only at 48 hpi in the saline-treated animals (Figures 4B,C). As mentioned before, the neutrophils are the first immune cells recruited to the infection site, being crucial for the early control of pathogen spreading (9). We suggest that the neutrophils are recruited to the infection site very early, controlling the CHIKV replication and dissemination through NETs release. However, this process is not very effective since the CHIKV is still able to reach the blood on consecutive days, inducing NETosis by peripheral blood neutrophils, thus resulting in the elevated systemic levels of NETs. The DNAse treatment abrogates the CHIKV local control, leading to early virus dissemination and proliferation into the blood. In agreement with our data, the systemic NETs induction with intravenous administration of LPS was capable of reducing myxoma virus infection in mouse liver cells, and the DNase abrogated this protection (36). In our work, we used IFNAR^−/−^ mice, which were described as a model of systemic viral infection (23), to demonstrate that the systemic NETs induction contributes to the viral load control *in vivo*. Interestingly, it has been widely described that, in rheumatoid arthritis, the neutrophils infiltrate the synovium, releasing NETs and consequently leading to damage in the joint tissues (37). Undoubtedly, chronic and debilitating arthralgia, accompanied by severe joint pain, is the most important consequence of a CHIKV infection. Thus, albeit we focused on the role of the NETs during an acute CHIKV infection, we do not discard the possibility of this mechanism in influencing chronic CHIKV infection.

Clinical studies have already reported that the virus-infected patients have an increased production of NETs (27, 34). In our study, we found that, similar to the mouse neutrophils, the human neutrophils isolated from healthy donors after *in vitro* incubation with CHIKV were able to release NETs (Figures 5A,B). Moreover, we had access to the serum of patients confirmed to be naturally infected with CHIKV (acute infections), in which we found a correlation between viremia and NETs level (Figures 5C,D), suggesting that the NETs were elevated due to a response against the increased viral load. Unfortunately, we were not able to access other clinical parameters (i.e., disease manifestation, evolution to chronic arthralgia) to associate the protective effect of NETs induction. Altogether our data support the role of NETs induction as a mechanism to control virus replication at early time points following an infection.

## Conclusion

Taken together, the results of our work are the first to demonstrate the protective role of NETs during an acute CHIKV infection. Additionally, the induction of NETs might be a useful tool to help in virus clearance, protecting the host and avoiding disease chronification.

## Data Availability Statement

All datasets generated for this study are included in the article/Supplementary Material.

## Ethics Statement

The studies involving human participants were reviewed and approved by Research Ethics Committee of Oswaldo Cruz Foundation, approval number (2.566.608). The patients/participants provided their written informed consent to participate in this study. The animal study was reviewed and approved by Caracterização das NETs em infecções por Arbovírus (5/2017)—Ethics Committee on the Use of Animals (CEUA) of the Ribeirão Preto Medical School, University of São Paulo.

## Author Contributions

CH, JT-K, MF, and DC performed the experiments and data analysis. CH, JT-K, MF, RF, and FC performed the manuscript preparation. FC, CH, JT-K, MF, LF, BF, and RF contributed to data interpretation and discussion. RF and FC conceived and supervised the study. All authors discussed the results and contributed to the final manuscript.

### Conflict of Interest

The authors declare that the research was conducted in the absence of any commercial or financial relationships that could be construed as a potential conflict of interest.
