# Supplementary material for: Neutrophil Extracellular Traps Effectively Control Acute Chikungunya Virus Infection
Source: Front Immunol. 2020 Jan 31;10:3108. doi: 10.3389/fimmu.2019.03108 (PMC7005923; doi:10.3389/fimmu.2019.03108)
Supplement: Supplementary file 1 [file Image_1.pdf]

Supplementary Material

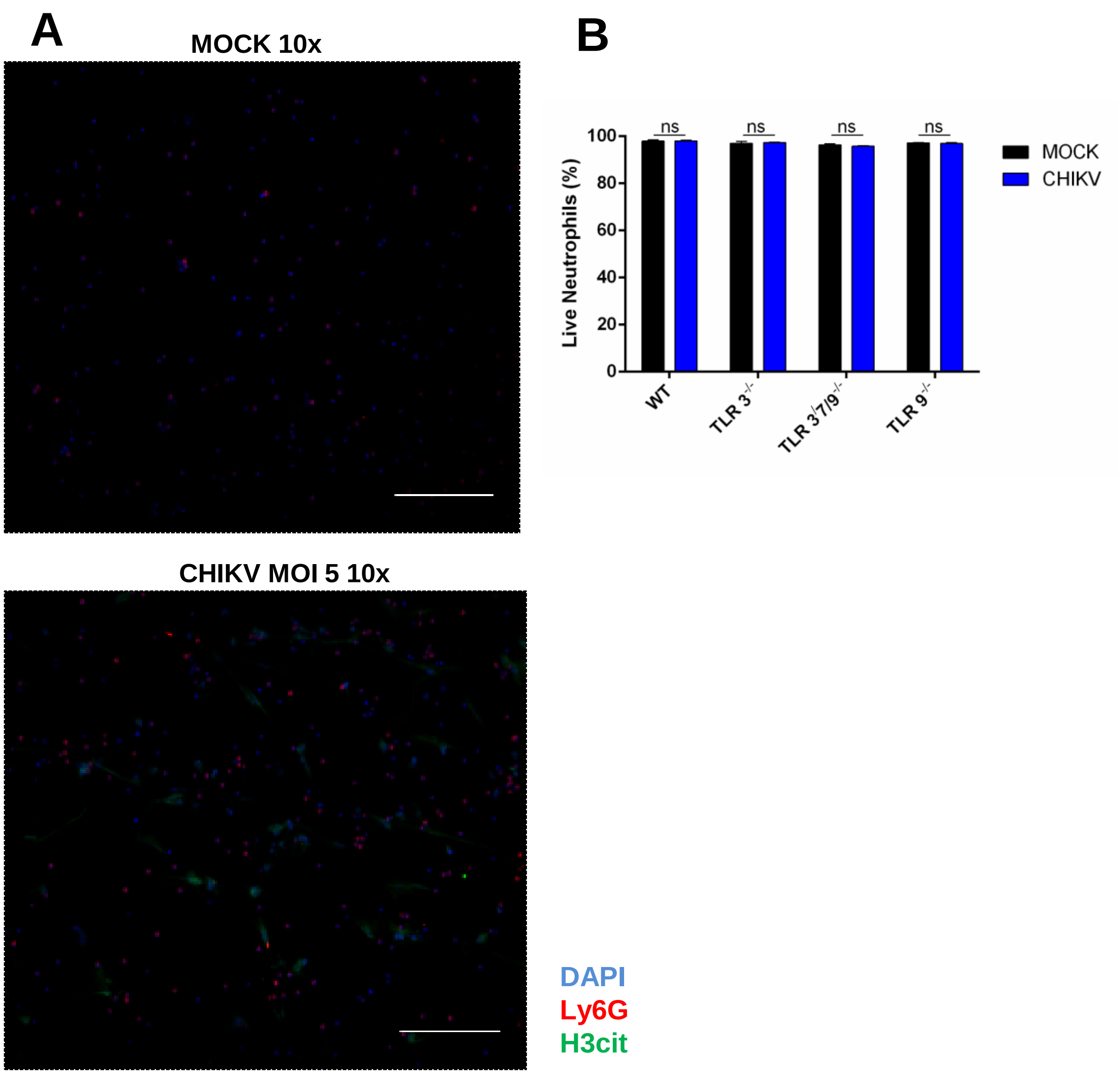

Supplementary FIGURE 1 (A) Representative image of immunofluorescence of mouse neutrophils incubated with CHIKV for 4 h. *Bars* = 250 µm. Magnification of ×10. Samples were stained with DAPI (*blue*), anti-Ly6G (*red*), and anti-H3 citrulline (*green*). **(B)** Percentage of live neutrophils isolated from WT, $TLR3^{-/-}$, $TLR3/7/9^{-/-}$, and $TLR9^{-/-}$ mice and incubated with MOCK control or CHIKV for 4 h. Data are presented as mean ± SD (n=2 per group). Statistical analysis was made by two-way ANOVA with Bonferroni's comparisons test. Representative results of one experiment performed independently
